# Exploring electrophysiological markers of auditory predictive processes and pathological ageing in adults with Down's syndrome

**DOI:** 10.1111/ejn.15762

**Published:** 2022-07-24

**Authors:** Chiara Avancini, Sally Jennings, Srivas Chennu, Valdas Noreika, April Le, Tristan A. Bekinschtein, Madeleine J. Walpert, Isabel C. H. Clare, Anthony J. Holland, Shahid H. Zaman, Howard Ring

**Affiliations:** ^1^ Cambridge Intellectual and Developmental Disabilities Research Group, Department of Psychiatry University of Cambridge Cambridge UK; ^2^ Cambridge Cognition Cambridge UK; ^3^ School of Computing University of Kent Canterbury UK; ^4^ Department of Biological and Experimental Psychology, School of Biological and Chemical Sciences Queen Mary University of London London UK; ^5^ Department of Psychology University of Cambridge Cambridge UK; ^6^ Cambridgeshire & Peterborough NHS Foundation Trust Cambridge UK

**Keywords:** ageing, auditory violation, dementia, MMN, P300, prediction error

## Abstract

Down's syndrome is associated with pathological ageing and a propensity for early‐onset Alzheimer's disease. The early symptoms of dementia in people with Down's syndrome may reflect frontal lobe vulnerability to amyloid deposition. Auditory predictive processes rely on the bilateral auditory cortices with the recruitment of frontal cortices and appear to be impaired in pathologies characterized by compromised frontal lobe. Hence, auditory predictive processes were investigated to assess Down's syndrome pathology and its relationship with pathological ageing. An auditory electroencephalography (EEG) global–local paradigm was presented to the participants, in which oddball stimuli could either violate local or higher level global rules. We characterised predictive processes in individuals with Down's syndrome and their relationship with pathological ageing, with a focus on the EEG event‐related potential called Mismatch Negativity (MMN) and the P300. In Down's syndrome, we also evaluated the EEG components as predictor of cognitive decline 1 year later. We found that predictive processes of detection of auditory violations are overall preserved in Down's syndrome but also that the amplitude of the MMN to local deviancies decreases with age. However, the 1‐year follow‐up of Down's syndrome found that none of the ERPs measures predicted subsequent cognitive decline. The present study provides a novel characterization of electrophysiological markers of local and global predictive processes in Down's syndrome.

## Graphical abstract

Down's syndrome (DS) is associated with pathological ageing characterised by early frontocentral impairment. We characterised frontocentral electrophysiological markers of local and global auditory prediction processes in DS. We assessed whether those markers may inform on pathological ageing in DS. Detection of local and global violations appears to be preserved in DS. The amplitude of the MMN elicited by local auditory violations was also a predictor of age in DS but not in controls.

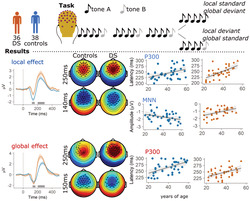

## 1 INTRODUCTION

People with Down's syndrome (DS) experience early deterioration of frontal cortices (Annus et al., 2016) as a result of amyloid overproduction caused by the triplication of the amyloid precursor protein gene, which is located on chromosome 21. In this population, early amyloid binding begins in the striatum, followed by the dorsal prefrontal cortex and the anterior cingulate cortex, which causes early onset of dementia characterised by executive symptoms (Annus et al., 2016; Zigman, 2013). Crucially, Alzheimer's disease (AD) pathology affects nearly the entirety of the DS population as they age with a prevalence of clinical dementia rising to over 40% by the time individuals reach their 50s and 75% by their 60s (Holland et al., 1998; Holland & Ball, 2009). While in sporadic and familial AD neuropathological changes begin years before symptoms onset (Sperling et al., 2013; Teipel et al., 2020), the time lag between amyloid deposition and cognitive decline is thought to be reduced in DS and the early symptoms may reflect a deterioration of frontal cortices (Annus et al., 2016; Ball et al., 2010, 2006, 2008). Given the tight relationship between DS pathology and pathological ageing, we investigated how evoked electrical brain activity can help lead to an understanding of the mechanisms through which DS neuropathology results in decline and how it progresses with ageing. Importantly, EEG has the advantage of being an inexpensive, non‐invasive and relatively participant‐friendly technology. Hence, EEG may be a particularly useful approach in research or in clinical practice when people with intellectual disabilities and/or people with dementia are involved.

Auditory predictive processes rely on the bilateral auditory cortices with the recruitment of frontal cortices (Doeller et al., 2003; Garrido et al., 2008; Giard et al., 1990; Liasis et al., 2001) and appear to be impaired in pathologies characterized by compromised frontal lobe (Alho et al., 1994; Hughes & Rowe, 2013; Näätänen et al., 2011; Pekkonen et al., 1996, 2001). In particular, compromised auditory predictive processes in patient with frontotemporal dementia have been linked to the compromised integrity of frontal and frontotemporal connections (Hughes & Rowe, 2013). Hence, the role of the frontotemporal network in auditory predictive processes has been supported by studies on clinical populations, but, to the best of our knowledge, no study has been conducted with participants with DS. Given the early frontal symptoms DS‐AD pathology (Ball et al., 2010, 2006, 2008), we identified markers of auditory predictive processing as potential informative markers of pathological ageing in people with DS, such as the auditory mismatch negativity (MMN) and the following P300.

The MMN is a negative deflection peaking around 150–200 ms after the presentation of violations of auditory regularities (Näätänen et al., 2004, 2007). The presentation of a stimulus that deviates in one or more of its features creates a mismatch between the incoming information and the memory trace of the regular stimuli, eliciting the MMN. According to the predictive coding framework (Friston, 2003, 2005), auditory predictive processes rely on a hierarchically organised cortical system that creates neural predictions about future events. The cortex generates top‐down predictions that are then compared with incoming sensory stimuli at the first level, or to a bottom‐up input at any higher level. If there is a discrepancy between the top‐down prediction and the bottom‐up input, a prediction error occurs and the MMN is generated (Garrido et al., 2008; Garrido, Kilner, Kiebel, & Friston, 2009; Garrido, Kilner, Stephan, & Friston, 2009). The generation of the MMN has also been attributed to pre‐perceptual change detection and the involuntary attention switch when the incoming stimulus deviates from the sensory memory trace in the auditory cortex (May & Tiitinen, 2007; Näätänen et al., 2007). Some studies suggest that in the typically developing (TD) population, the amplitude of the MMN is attenuated in older compared with younger individuals (Cheng et al., 2013; Horváth et al., 2007; Kiang et al., 2009; Näätänen et al., 2011; Schiff et al., 2008). On the other hand, a recent study specifically assessing auditory predictive processes in the TD senior population did not find a decrease in MMN response with age (Hsu et al., 2021).

A crucial feature of the MMN response is that elicited independently of the participant's direction of attention (Näätanen et al., 1978, 2007), which makes it a suitable measure for people with DS who may have impaired attention (Lott & Dierssen, 2010) and may struggle to attend to stimuli during a testing session. The topography consistent with the locus of early neurophathology in DS and its disengagement from attentional processes were key characteristics evaluated when choosing the MMN to investigate the progression of dementia in people with DS.

Along with the MMN, rare events also elicit a P300 response which is a positive deflection peaking between 250 and 400 ms after stimulus onset and that is a marker of the perception of salient events and their encoding in working memory (Sutton et al., 1967). Specifically, two separate positive components have usually been observed in response to oddball events: The centro‐frontal P300 peaking around 250–300 ms is elicited by novel stimuli even if those are irrelevant to the task. This often follows the MMN marking a bottom‐up reorienting of attention (Donchin, 1981; Escera et al., 2001; Polich, 2007); the centro‐parietal P300 peaking around 300–350 ms reflects top‐down selective attention to task‐relevant stimuli, their encoding in working memory and entry in conscious awareness (Kok, 2001; Polich, 2007). Within the predictive coding framework, the parietal P300 reflects a residual prediction error in the case of longer term stimulus deviance that require stimulus awareness (Chennu et al., 2013; Chennu & Bekinschtein, 2012). The P300 and its components have been found to decrease in amplitude and increase in latency with normal ageing, as well as being able to distinguish controls to individual with AD (Cecchi et al., 2015; Polich et al., 1990; Polich & Corey‐Bloom, 2005). Research on the P300 in people with DS is surprisingly scarce and outdated. Overall, the limited literature available suggests that P300 latencies are longer and amplitudes smaller in people with DS than TD controls, with contrasting evidence of the P300 being a potential marker of ageing in this population (Blackwood et al., 1988; César et al., 2010; Kakigi et al., 1994; Medaglini et al., 1997; Muir et al., 1988).

The auditory MMN/P300 complex is usually followed by the reorienting negativity (RON). The RON is thought to index the recovery from distraction by reorienting attentional resources towards task‐relevant stimuli (Justo‐Guillén et al., 2019). The RON to local deviances has been found to be absent in seniors (Hsu et al., 2021). Furthermore, it appears to be reduced in patients with chronic alcoholism, chronic schizophrenia, unmedicated Parkinson's disease, and amyotrophic lateral sclerosis (Higuchi et al., 2014; Polo et al., 2003; Rissling et al., 2012; Solís‐Vivanco et al., 2011; Volpato et al., 2016). Along with the MMN and the P300, it is also proposed as a measure of frontal lobe integrity. However, in comparison to the MMN/P300 complex, the RON has been scarcely explored neither in neuropsychological disorders or TD subjects (Horváth et al., 2008; Justo‐Guillén et al., 2019).

On the basis that frontal and supratemporal cortices contribute to the MMN and P300 generation and that people with DS have an increased vulnerability to frontal lobe dysfunction, we hypothesised that changes across age in the markers of violations detection may reflect pathological ageing and would be associated with age‐related cognitive decline in people with DS. In this study, participants with DS and age‐matched TD controls were presented a global–local paradigm during EEG recording in which frequent and deviant tones were presented. The paradigm was designed to engage both bottom‐up and top‐down stimulus processing. Participants were also assessed using neuropsychological tests sensitive to age‐related cognitive decline. After 1 year, the same DS participants were re‐administered the neuropsychological test battery to determine the presence of cognitive decline.

Given the paucity of studies on the MMN and the P300 in participants with DS, our first aim was to characterise these components in the DS population. Our second aim was to determine whether markers of violation detection could detect pathological ageing in DS. Specifically, we hypothesized that increasing age would result in smaller MMN and P300 amplitudes and longer latencies in both groups but with a stronger effect in DS compared with TD participants. Furthermore, we hypothesized that the status of the ERP components at the first time‐point would predict future cognitive decline in those participants with DS as measured by comparison in neuropsychological test scores between the two time points 1 year apart. Given consistent differences in the head shape of those with DS compared with TD, we used data driven approaches to localize the ERP components in TD and DS groups.

## 2 MATERIALS AND METHODS

The present study was conducted at two time points (T1 and T2). A case–control study was conducted at T1 in which DS and aged‐matched TD participants took part to the EEG session. A year later at T2, only DS participants were re‐tested on neuropsychological tests to determine whether there had been cognitive decline since T1.

Ethical approval to conduct the study (reference 14/LO/1411) was given by the National Research Ethics Service (NRES). The Committee had the expertise to assess studies that might include individuals who lacked capacity to consent to participation in research.

### 2.1 Participants at T1

Thirty‐six adults with DS aged 22–55 years (*M* = 37.3, *SD* = 9.39, 21 males; see Table 1) were recruited into the study. Participants with DS were predominantly identified through their previous participation in the ‘Defeat Dementia in Down's Syndrome’ research programme. Participants who were not already known to the research group were made aware of the study through information from the Down's Syndrome Association. Thirty‐eight TD controls aged 20–59 years old (*M* = 39.84, *SD* = 11.40, 17 males; see Table 1) were recruited into the study through the Join Dementia Research (JDR) database. Ethical approval to conduct the study (reference 14/LO/1411) was given by the National Research Ethics Service (NRES). The Committee had the expertise to assess studies that might include individuals who lacked capacity to consent to participation in research.

**TABLE 1:** Participants' demographics. Sex, age, and hearing acuity scores of the two groups

	*N*	Males	Females	Mean age (years)	*SD*	Age range	Mean number of tones heard	*SD*
DS	36	21	15	36.81	9.22	22–55	9.83	1.8
Controls	39	17	22	39.84	11.40	20–59	10.33	0.70

### 2.2 Participants at T2

Thirty‐five adults with DS who completed the initial EEG assessment were re‐approached 10–14 months later (mean 12 months) for a follow‐up cognitive assessment. One participant was not re‐approached because during the cognitive assessment carried out at T1 he had not been able to co‐operate with the assessment.

### 2.3 Clinical assessments

#### 2.3.1 Hearing checks

As the EEG paradigms involved the presentation of auditory stimuli, hearing loss was screened for with the Siemens HearCheck Navigator, which has been validated as an appropriate tool (Fellizar‐Lopez et al., 2011). This portable screener was taken to the homes of participants with DS. The aim of this was to reduce participant burden (i.e., unnecessary travel to site for EEG assessment). The HearCheck Navigator sequentially delivered tones at two frequencies (1000 Hz, 3000 Hz) and a range of decibels (20–75 dB). First, three 1000‐Hz sounds were presented at 55, 35, and 20 dB. Then, three 3000‐Hz sounds were presented at 75, 55, and 35 dB. The procedure was performed for each ear for a total of 12 sounds presented. Participants who did not hear tones of 1000 and 3000 Hz at 55 dB were to be excluded from the study and they were not brought to site. No one with this degree of hearing loss was identified.

#### 2.3.2 Intellectual functioning

The Kaufman Brief Intelligence Test‐2 (KBIT‐2; Kaufman & Kaufman, 2014) was used to estimate intellectual functioning in both groups. The KBIT‐2 provides verbal (VIQ) and nonverbal IQ (NIQ) scores. Normally, these are to produce a Composite IQ score. Where the VIQ and NIQ discrepancy is too large (see Table B 7; Kaufman & Kaufman, 2014), Verbal IQ on its own is used.

#### 2.3.3 Dementia screening in TD

The Addenbrooke Cognitive Examination Revised (ACE‐R; Mioshi et al., 2006) was used to screen control participants for dementia. Following the cut‐off of 88 or below, no control participants were excluded from this study based on their ACE‐R assessment.

#### 2.3.4 Cognitive decline in DS

To make a diagnosis of dementia and to track cognitive decline in DS, The Cambridge Examination for Mental Disorders of Older People with Down's Syndrome and Others with Intellectual Disabilities CAMDEX‐DS (CAMDEX‐DS; Fonseca et al., 2018; Holland & Ball, 2009; Roth et al., 1986) was administered at the assessments at T1 and T2. The CAMDEX‐DS was developed as a tool to aid the diagnosis of dementia in people with intellectual disability. It includes a cognitive assessment component (CAMCOG‐DS) assessing functional domains affected by the presence of AD and an informant interview (CAMDEX‐DS). The CAMDEX‐DS informant interview was used to identify functional decline and to structure the diagnosis of dementia in people with DS based on reported change across specific functional domains. In this study, the diagnosis of dementia in participants with DS was made by an experienced psychiatrist reviewing, blind to the age, gender, and previous diagnostic status of the participant, the data collected using the CAMDEX‐DS informant interview with the parent or carer.

### 2.4 EEG assessment

#### 2.4.1 Paradigm

A modified version of the auditory global–local paradigm (Bekinschtein et al., 2009) was used, previously described in Chennu et al. (2013). The global–local paradigm was designed to measure prediction error responses at two hierarchical levels of deviation: (i) Global—between trial variance determined by the presentation of rare sequences of tones which elicit an attention‐dependent responses; (ii) Local—within trial variance determined by the automatic detection of individual deviant tones that elicits bottom‐up processes even in absence of attention (Chennu et al., 2013).

**FIGURE 1:**
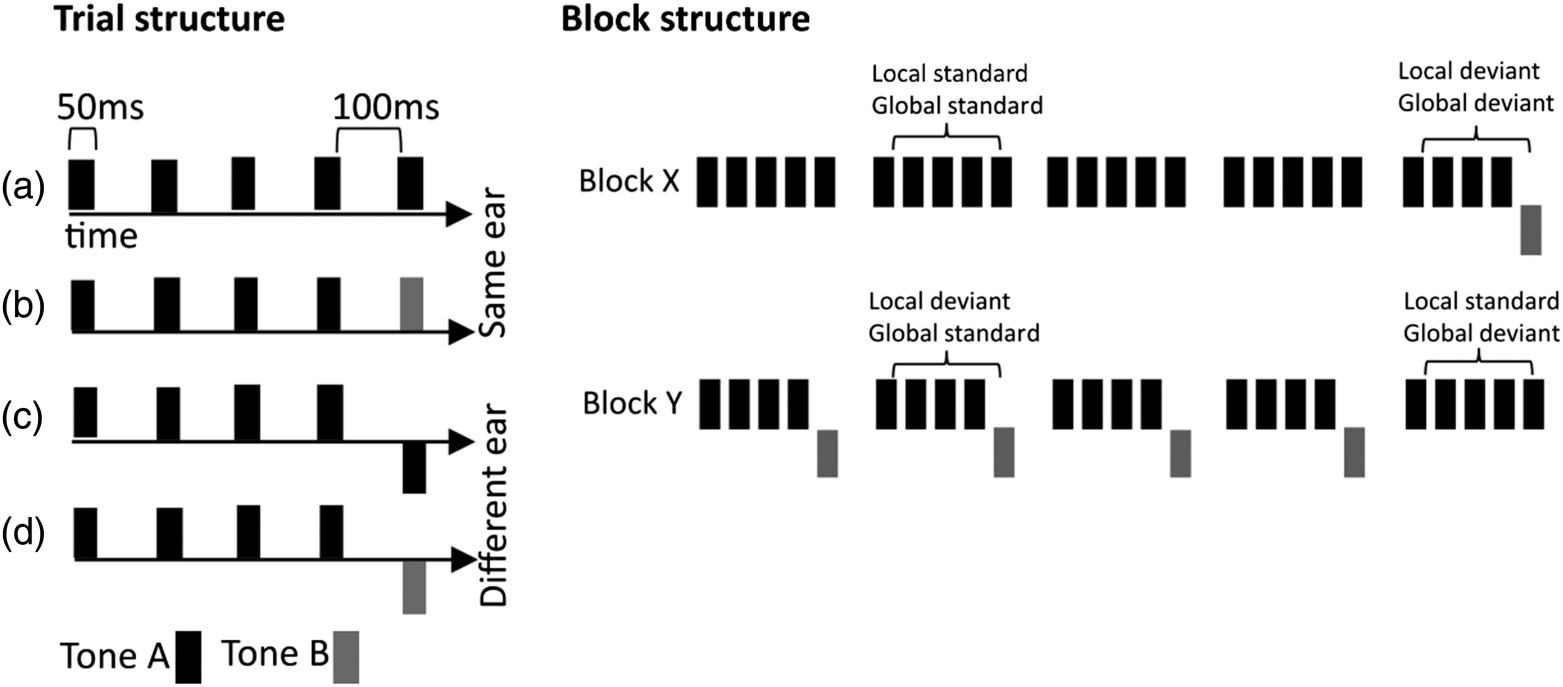
Left panel: Description of trial structure. Trials were composed of a five tones sequence. In *standard* sequences, tones were all of the same pitch and presented monoaurally in the same ear (a). In *deviant* sequences, the fifth tone could either be of a different pitch (b), presented in the opposite ear (c) or both of a different pitch and presented in the opposite ear (d). Tones were 50 ms long and were spaced by 100 ms silence. Right panel: Example of X and Y blocks that allowed orthogonal contrasts between local and deviant sequences. In X trials, local standards appeared 85.75% of the times therefore being global standards. In Y trials, local standards appeared 14.25% of the times therefore being global deviants.

The paradigm consisted of the presentation of tones at a volume that the participants indicated as audible and comfortable. Each tone lasted 50 ms and was presented in group sequences of five tones with 100‐ms intervals in between each tone. The five‐tones group consisted of sequences in which five tones had identical pitch (AAAAA or BBBBB), or sequences in which the first four tones were identical and the last tone had a different pitch (AAAAB or BBBBA). The tones themselves were mixtures of three sinusoids of either type: A (500, 1000, and 2000 Hz), or B (350, 700, and 1400 Hz). The tone sequences were presented either entirely monaurally (AAAAA, BBBBB, AAAAB, BBBBA), to the left or right ear, or predominantly monoaurally with the final tone presented on the opposite ear (AAAA*A*, BBBB*B*, AAAA*B*, BBBB*A*). Sequences could be grouped in *local standard*, in which five identical tones were presented monoaurally (AAAAA or BBBBB), or *local deviant*, in which the last tone was either of a different pitch, or was presented in the opposite ear, or deviated in both pitch and ear of presentation (AAAAB, BBBBA, AAAA*A*, BBBB*B*, AAAA*B*, BBBB*A*; see Figure 1). Tone sequences were presented in experimental blocks, and each block included approximately 160 sequences and was counterbalanced by the dominant tone type (A or B) and the laterality of monoaural tone delivery (left or right). Furthermore, in each block, there were whole sequences that were frequent (*global standard*) and sequences presented less frequently (*global deviant*). *Global deviant* sequences were pseudorandomly interspersed among *global standards*. There were always between two to five *global standard* sequences in between *global deviant* sequences. There were two block types (Figure 1). In block type X, *local deviant* sequences were 28.5% of the total sequences, the rest being local standards (i.e., local standards were also global standards). In block type Y, *local deviant* sequences were 71.5% of the total sequences (i.e., local deviants were global standards). The two block types allowed to create orthogonal contrasts between the local and global tone deviance.

At the beginning of the testing session, participants were informed that they were about to hear groups of sounds. Participants were asked to listen carefully to the groups of sounds because at the end of each block they would be asked: “Can you tell me what group of sounds you heard a lot?” and “can you tell me what group of sounds you heard sometimes?” Participants' answers were recorded at the end of each block. The purpose of the questioning was to maintain participants' attention on the groups of sounds in order to assess the global effect. At the end of each block, participants were also asked about their arousal levels on a scale of 1–10 (1 = Asleep to 10 = Fully awake), and attentiveness (1 = Mind wandering/unattentive to 10 = Fully attentive to stimuli). Participants took a break between each block at a length of their choosing. A total of eight experimental blocks (four Y blocks and four X blocks) were presented, with total testing time, including breaks and questioning, taking an average of 40 min.

#### 2.4.2 Data acquisition

Data were recorded with 129‐channels EEG gel nets (EGI's HydroCel Geodesic Sensor Net). Testing was conducted in an electrically shielded room, using the Net Amps 300 amplifier (Electrical Geodesics, Inc.). The auditory stimuli were presented to participants using Psychtoolbox‐3 (Kleiner et al., 2007) running on MATLAB R2015b. They were played binaurally through binaurally through Etymotics ER‐3A earphones. They were presented with an intensity of 70 dB and some participants requested minor adjustment. The EEG data were recorded with Net Station Version 5.4 (Magstim EGI). The recording parameters for collection were as follows: <10‐kΩ impedance, 500‐Hz sampling rate, and a vertex reference.

#### 2.4.3 Data preprocessing

Pre‐processing was run in MATLAB R2019b using custom functions along with EEGLAB toolbox v13.5.4b (Kleiner et al., 2007). Channels of the outer circle of the net were excluded from the pre‐processing as they carry little neural information and mostly muscle artifacts (Chennu et al., 2013). Continuous data were low‐pass filtered offline at 20 Hz (Garrido et al., 2008; Näätänen et al., 2004, 2005). The data were epoched relative to the presentation of the fifth tone, which occurred at 550 ms after the start of the tones sequence. Epochs were selected from −200 to 700 ms relative to the onset of the fifth tone in each sequence and baseline corrected −200 to 0 ms relative to the fifth tone onset. Bad electrodes were detected by a quasi‐automated procedure: Noisy channels were identified by calculating their normalized variance and then manually rejected or retained by visual inspection. Rejected channels were excluded from ICA decomposition, which was used to remove eyeblinks and lateral saccades. Robust detrending was applied on the ICA corrected signal to correct for slow drifts. Subsequently, rejected channels were interpolated and epochs exceeding ±150 μV were marked and then discarded following further visual inspection and the signal was then re‐referenced to the average. An average of 6% of the trials were rejected (range = 0–36%). For both global and local effects, *standard* and *deviant* trials were pooled across X and Y blocks. For the local effect, the ERP was obtained for *local standard* and *local deviant* (grouping all deviant types) conditions and the *difference wave* was obtained by subtracting the waveform of *local standard* epochs from the waveforms of *local deviant* epochs and were baseline corrected after the subtraction. Similarly, for the global effect the ERP was obtained for *global standard* and *global deviant* conditions and the *difference wave* was obtained by subtracting the waveform of *global standard* epochs from the waveforms of *global deviant* epochs. Baseline correction was performed after the subtraction. Before averaging, the number of epochs contributing to a participants' *standard* and *deviant* ERPs were equalized across conditions. For the analysis of the local effect, an average of 473.06 (*SD* = 10.81) epochs per participant were retained in the TD control group and an average of 428.06 epochs per participant (*SD* = 43.62) in the DS group. For the analysis of the global effect an average of 252.16 (*SD* = 4.82) epochs per participant were retained in the TD control group and an average of 226.64 (*SD* = 24.01) epochs per participant in the DS group. Because the number of epochs was equalized across conditions, the descriptive statistics refer to the number of epochs in each individual condition (e.g., an average of 473.06 local standard and 473.06 local deviant epochs retained in the TD group). Participants were excluded if after trials rejection less than 100 epochs were retained in any condition. No participant met this criterion.

**FIGURE 2:**
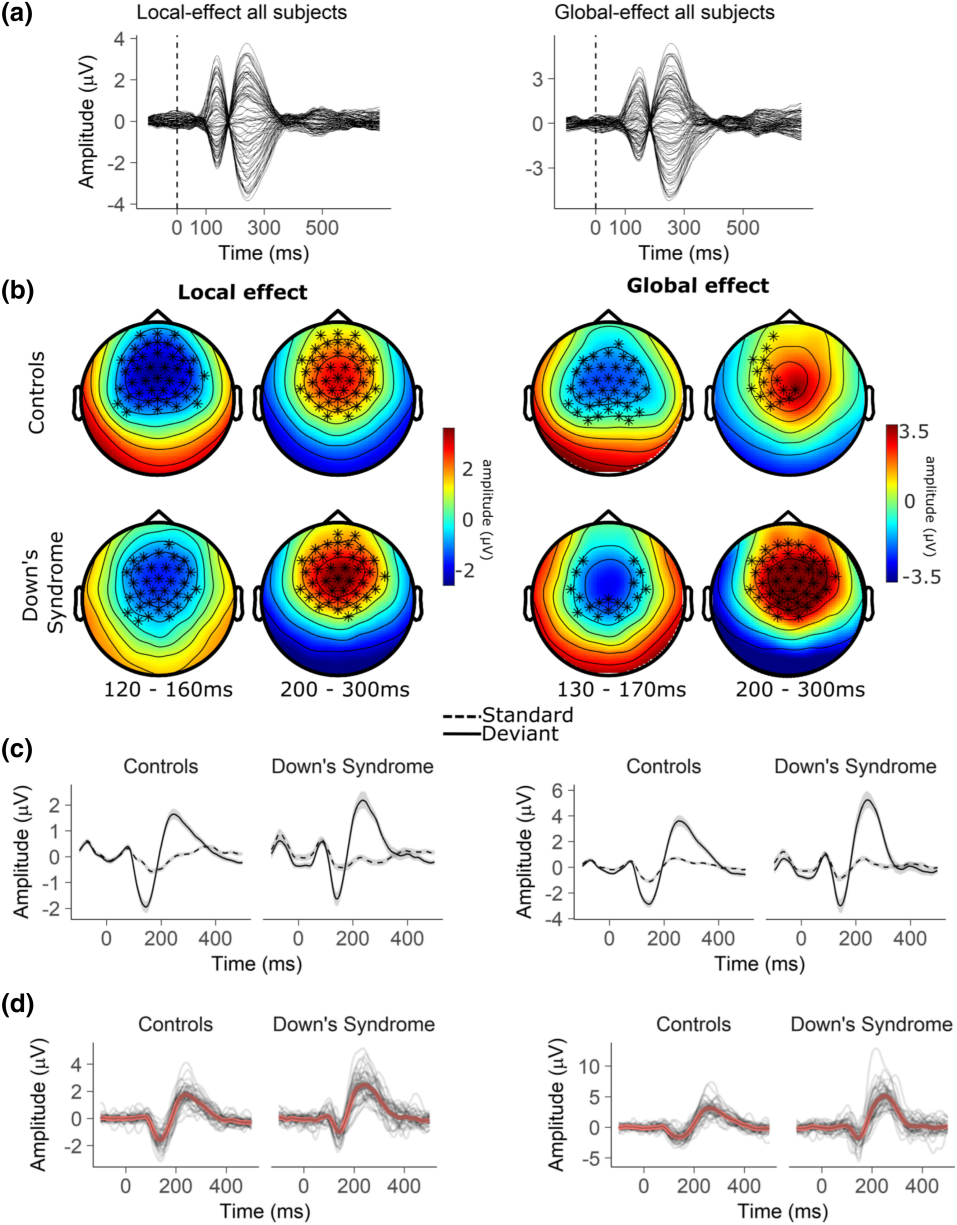
(a) Butterfly plot displaying the grand average of the MMN difference wave at all electrodes. Both local and global effects are displayed. The plot represents data of DS and TD controls merged together. (b) Results of the cluster permutation analysis contrasting the *standard* and *deviant* conditions for both local and global effects in the relevant time‐window of the lMMN and lP300 (left), and the gMMN and gP300 (right). The electrodes of the significant clusters (*α* ≤ 0.01) are marked in bold. The colour bar represents the amplitude of the difference wave obtained subtracting the waveform elicited by the *standard* condition to the waveform elicited by the *deviant* condition. (c) Depiction of the local (left) and global (right) ERPs in the *standard* and *deviant* conditions in both groups. (d) Difference waveforms of the local (left) and global (right) effects. The red waveforms represent the grand averages and the thin black waveforms each individual participant.

Because only a very few studies have characterised the ERP in people with DS the peak of the components of interest were initially identified by the inspection of butterfly plots where all participants were grouped together (Figure 2a). The MMN of the local effect (lMMN) was established as occurring at 140 ms after stimulus onset, the P300 of the local effect (lP300) at 250 ms, the MMN of the global effect (gMMN) at 150 ms, and the P300 of the global effect (gP300) at 250 ms. In order to select the relevant electrodes for ERP analysis for each group, the cluster‐permutation algorithm (FieldTrip toolbox; Maris & Oostenveld, 2007) was run separately for TD controls and DS contrasting *deviant* and *standard* trials both for the local and global effects. Two‐tailed dependent *t*‐tests were used to evaluate the effect. At the cluster level, the null hypothesis distribution was generated using the Monte Carlo method and the critical value used for thresholding the sample‐specific *T*‐statistics was set at *α* = 0.01. The relevant electrodes selected for each component were those forming significant clusters within the following time windows: 120–160 ms for the lMMN, 120–170 ms for the gMMN, and 200–300 ms for the lP300 and the gP300 (Figure 2b, electrodes marked in bold). Peak detection was performed on the average of the selected electrodes for each component. For each individual participant, the peaks corresponding to the lMMN and the gMMN were defined as the most negative value within the respective time windows. The peaks corresponding to the lP300 and gP300 were defined as the most positive value within the respective time windows. Both amplitudes and latencies were extracted for statistical analyses.

### 2.5 Control–case study statistical analyses

**FIGURE 3:**
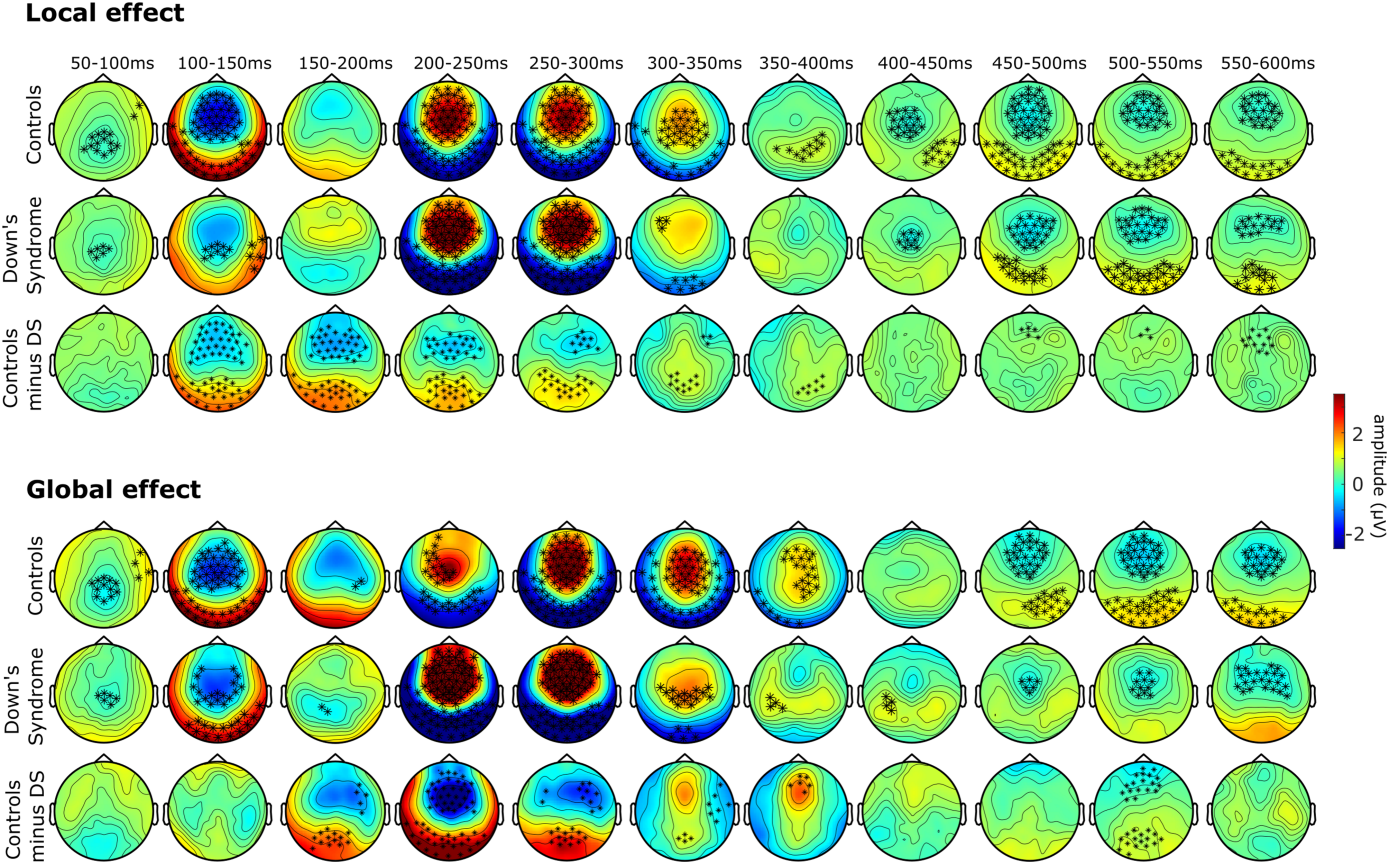
Time course of the local (top) and global (bottom) effects between 50 and 600 ms of TD controls, DS and the difference between the two groups. Electrodes of significant clusters (*α* ≤ 0.01) are marked in bold.

To evaluate the time course of the local and global effects in both groups, *deviant* and *standard* trials were contrasted with two‐tailed dependent *t*‐tests in a cluster permutation analysis run from 50 to 600 ms after tone onset. Similarly, the time course of the group difference (TD minus DS) in local and global responses was assessed with cluster permutation analysis on the difference waves and using two‐tailed independent *t*‐tests. The results of the analysis on the time course of the effects are summarised in consecutive 50 ms time windows (Figure 3).

At the cluster level, the null distribution was generated using the Monte Carlo method and the critical value used for thresholding the sample‐specific *t*‐statistics was set at *α* = 0.01.

The Shapiro–Wilk test for normality was run on amplitude and latency of all components to check whether they were normally distributed. To test whether the components differed between the two groups, amplitudes and latencies were compared using two‐tailed independent samples *t*‐tests or the Wilcoxon rank sum test in the case of not‐normally distributed data. In the case of a significant difference, two‐samples comparisons were run on participants younger than 40 years of age to test whether differences in ERP were present already in young participants. For all components, multiple regressions were fitted to the data and hierarchical model comparison was conducted by means of likelihood ratio tests (*lrtest()* in R) to assess if age predicted changes in ERP in the two groups. Likelihood ratio tests compare hierarchically nested models to determine if adding complexity to the model improves its fit. Rejecting the null hypothesis provides evidence for accepting the more complex model. Models were built with amplitude and latency as dependent variables, and with the factors *Group* and *Age* as predictors (models in Formulae 1). Models *a* to *d* were compared in the order exposed in Formulae 1. Models were also compared calculating their Bayes factors (*BF*) with 50% prior (*bayesfactor_models()* in R). In case of a significant *Group* and *Age* interaction, linear models for the two separate groups were fitted and contrasted to the intercept (models *e* and *f* in Formulae 1).

Through likelihood ratio tests and Bayesian model comparison we could determine the model that best explained the data. For the best model we reported F statistics and adjusted *R* squared (*R* ^2^_Adj_) to describe the fit of the model. We also reported each of its predictors' contribution.

-
y~1
-
y~Age
-
y~Age+Group
-
y~Age*Group
-
$$y_{\text{group}}\sim {\mathrm{Age}}_{\text{group}}$$
-
$$y_{\text{group}}\sim 1$$

Formulae 1 linear models were fitted and compared to assess whether age predicted changes in MMN across the two groups. *Y* is either amplitude or latency. Model (a) is the intercept model; model (b) expresses the relationship between physiological measures and *Age* as only predictor; model (c) includes the main effects of predictors *Age* and *Group*; model (d) includes the main effects of the predictors *Age* and *Group* as well as their interaction; model (e) expresses the relationship between the dependent variable of only one group (controls or DS) and age; and model (f) is the intercept model of data from only one group.

To account for the statistics been run on both amplitude and latency, the critical value was set to *α* = 0.025.

### 2.6 Statistical analyses to assess cognitive decline in DS

The analyses focused on the difference between participants' CAMCOG‐DS scores at T1 and T2 The total CAMCOG‐DS difference score (dCAMCOG) was calculated as the score at T2 minus the score at T1. To test whether performance at the CAMCOG at T2 decreased significantly compared with the performance at T1, a paired *t*‐test was run on CAMCOG scores at the two time points. To assess whether changes in CAMCOG‐DS scores over time correlate with age, a two‐tailed Spearman's rank‐order correlation was run between age and dCAMCOG. The Spearman test was chosen because research has suggested that the relationship between age and amyloid deposition is not linear (Holland et al., 1998). The BF was calculated for such correlation as well.

Finally, we tested whether ERPs amplitude and latency at T1 predicted changes in CAMCOG‐DS scores over time. We built models with *amplitude* and *latency* as predictors, *IQ* as covariate and dCAMCOG scores as dependent variable. To assess the relationship between variables, linear and curvilinear quadratic models (Formulae 2) were compared using likelihood ratio tests and BF were calculated. If a fitted model predicted dCAMCOG scores, the model was fitted to the scored to the CAMCOG‐DS subscales.

-
dCAMCOG~1
-
dCAMCOG~x
-
dCAMCOG~x+IQ
-
$$\text{dCAMCOG}\sim x+x^{2}+\mathrm{IQ}$$
-
$$\text{dCAMCOG}\sim x+x^{2}$$

Formulae 2 linear (a,b,c) and curvilinear (d,e) models compared to assess whether physiological measures predicted changes in CAMCOG scores (total score and subscales) over time in DS. *X* is either the amplitude or latency of the MMN.

Statistical analyses were run using R 4.4.0 (R Core Team, 2020).

## 3 RESULTS

### 3.1 Demographics at T1

Independent samples *t*‐tests for Equality of Means were conducted, with Equality of Variances assumed (*p* > 0.05) to determine that DS and TD controls did not significantly differ in age (*t*_70.49_ = 1.04, *p* = 0.15). Equality of Variance was not assumed (*p* ≤ 0.05) for group comparisons on hearing acuity, which was determined using the number of tones identified from the Siemens Hear Check Screener. However, an independent samples *t*‐test for the Equality of Means found that the number of tones identified did not significantly differ between groups (*p* = 0.12; Table 1). A chi‐square test of independence was performed to examine the relationship between gender (male, female) and group (DS, controls) and found no significant relationship: *χ* ^2^_75_ = 2.24, *p* = 0.13.

Of the 36 DS adults, three had a diagnosis of dementia. The participants' total CAMCOG score ranged from 55 to 105 points (*M* = 83.1, *SD* = 13.7). For four participants, KBIT‐2 Composite IQ scores could not be used; Verbal IQ scores ranged from 70 to 80 points (*M* = 77, *SD* = 4.7). For the remaining 32 participants, KBIT‐2 Composite IQ scores ranged from 40 to 88 points (*M* = 53.7, *SD* = 12.3). In the TD control group, the lowest Composite IQ score was 90 (*M* = 115.76, *SD* = 22.27); the lowest dementia‐screening score, as assessed by the ACE‐R, was 88. Therefore, the group is considered to be appropriate as TD controls for this study.

### 3.2 Local and global effects

Inspection of butterfly plots showed that the local effect manifested as two consecutive deflections peaking at 140 ms and at 250 ms (Figure 2a). In both groups, cluster‐based permutation analysis showed (*p* = 0.00010) the earlier deflection to be a frontocentral negativity (lMMN) and the latter to be a frontocentral positivity (lP300; Figure 2b,c). The Shapiro–Wilk test run on the peaks of the lMMN confirmed that both amplitudes and latencies were normally distributed. Peak amplitudes in participants with DS (*M* = −1.14 μV, *SD* = 0.62) were smaller than in controls (*M* = −1.71 μV, *SD* = 0.67; *t*_71.90_ = −3.80, *p* = 0.0030, Cohen's *d* = −0.88) and we did not find evidence for a difference between the two groups in young individuals (*t*_30.74_ = −0.94, *p* = 0.37). The computed *BF*_10_ = 0.45 suggests anecdotal evidence for the null hypothesis. *t*‐tests run on latencies did not show any significant difference between groups (DS: *M* = 140.4 ms, *SD* = 8.7; TD: *M* = 136.7 ms, *SD* = 9.4; *p* = 0.082). For the peaks corresponding to the lP300, the distribution of amplitudes and latencies only approached normality (*p*_amp_ = 0.030, *p*_lat_ = 0.041). Peak amplitude in participants with DS (*M* = 2.88 μV, *SD* = 1.07) was greater than in controls (*M* = 2.09 μV, *SD* = 0.77; *W* = 385, *p* = 0.0010, *r* = 0.38). Comparing the two groups only when individuals were below 40 years of age was there no evidence of a significant difference (*W* = 112, *p* = 0.052). However, the effect approached significance and a *BF*_10_ = 1.45 showed anecdotal evidence for the alternative. The Wilcoxon sum rank test run on latencies did not show any significant difference between groups (DS: *M* = 246.05, *SD* = 24.84; TD: *M* = 244.11, *SD* = 25.36).

The time course of the local effect was similar in the two groups (Figure 3). In TD controls the violation of local regularities generated three negative clusters between 50 and 150 ms (*p* = 0.00010), 200 and 350 ms (*p* = 0.00010), and 400 and 600 ms (*p* = 0.00010) and a positive cluster between 200 and 600 ms (*p* = 0.00010). In participants with DS, two significant negative clusters appeared between 50 and 150 ms (*p* = 0.00010) and 400 and 600 ms (*p* = 0.00010) and three positive clusters at 100–150 ms (*p* = 0.0060), 200–350 ms (*p* = 0.00010), and 400–600 ms (*p* = 0.00010). The comparison of the two groups showed two negative clusters between 50 and 350 ms (*p* = 0.00010) and 450and 600 ms (*p* = 0.0040) and a positive cluster between 50 and 400 ms (*p* = 0.00010).

Butterfly plots showed that the global effect manifested as two consecutive deflections peaking at 150 ms and at 250 ms (Figure 2a). In both groups, cluster‐based permutation analysis showed (*p* = 0.00010) the earlier deflection to be a frontocentral negativity (gMMN) and the latter to be a frontocentral positivity (gP300; Figure 2b,c). The Shapiro–Wilk test confirmed that amplitudes of the gMMN were normally distributed, while latencies were not (*p* = 0.00094). Independent *t*‐test and the Wilcoxon sum rank test found no differences between the two groups neither in amplitudes (DS: *M* = −1.88, *SD* = 0.87; TD: *M* = −1.66, *SD* = 0.67) nor latencies (DS: *M* = 146.67, *SD* = 10.59; TD: *M* = 148.26, *SD* = 14.54). Amplitudes of the gP300 were not normally distributed (*p* = 0.00044). The Wilcoxon sum rank test and the *t*‐test showed differences between groups amplitudes (*W* = 271, *p* = 0.0000034, *r* = 0.51) with the DS group having more positive peaks than TD controls (DS: *M* = 4.24, *SD* = 1.60; TD: *M* = 2.69, *SD* = 0.96) as well as shorter latencies (*t*_70.12_ = 3.40, *p =* 0.0011, Cohen's *d* = 0.79; DS: *M* = 246.89, *SD* = 20.12; TD: *M* = 264.84, *SD* = 25.09). Group differences in amplitudes (*W* = 59, *p* = 0.00026, *r* = 0.57) and latencies (*t*_26.15_ = 2.16, *p* = 0.039, Cohen's *d* = 0.74) were apparent already in participants below 40 years of age.

Violation of global regularities (Figure 3) in TD controls showed two significant negative clusters between 50 and 200 ms (*p* = 0.00010) and 450 and 600 ms (*p* = 0.0020) and three positive clusters between 50 and 150 ms (*p* = 0.00010), 200 and 400 ms (*p* = 0.00010), and 450 and 600 ms (*p* = 0.00010). In the DS group significant negative clusters emerged between 50 and 150 ms (*p* = 0.00010) and 450 and 600 ms (*p* = 0.0090), and two significant positive clusters between 100 and 150 ms (*p* = 0.0040) and 200 and 400 ms (*p* = 0.00010). Comparing the two groups, two significant negative clusters emerged between 150 and 350 ms (*p* = 0.00010) and 500 and 550 ms (*p* = 0.0050) and two positive clusters between 150 and 400 ms (*p* = 0.00010) and 500 and 550 ms (*p* = 0.0040).

### 3.3 Age as ERPs predictor

#### 3.3.1 lMMN

**FIGURE 4:**
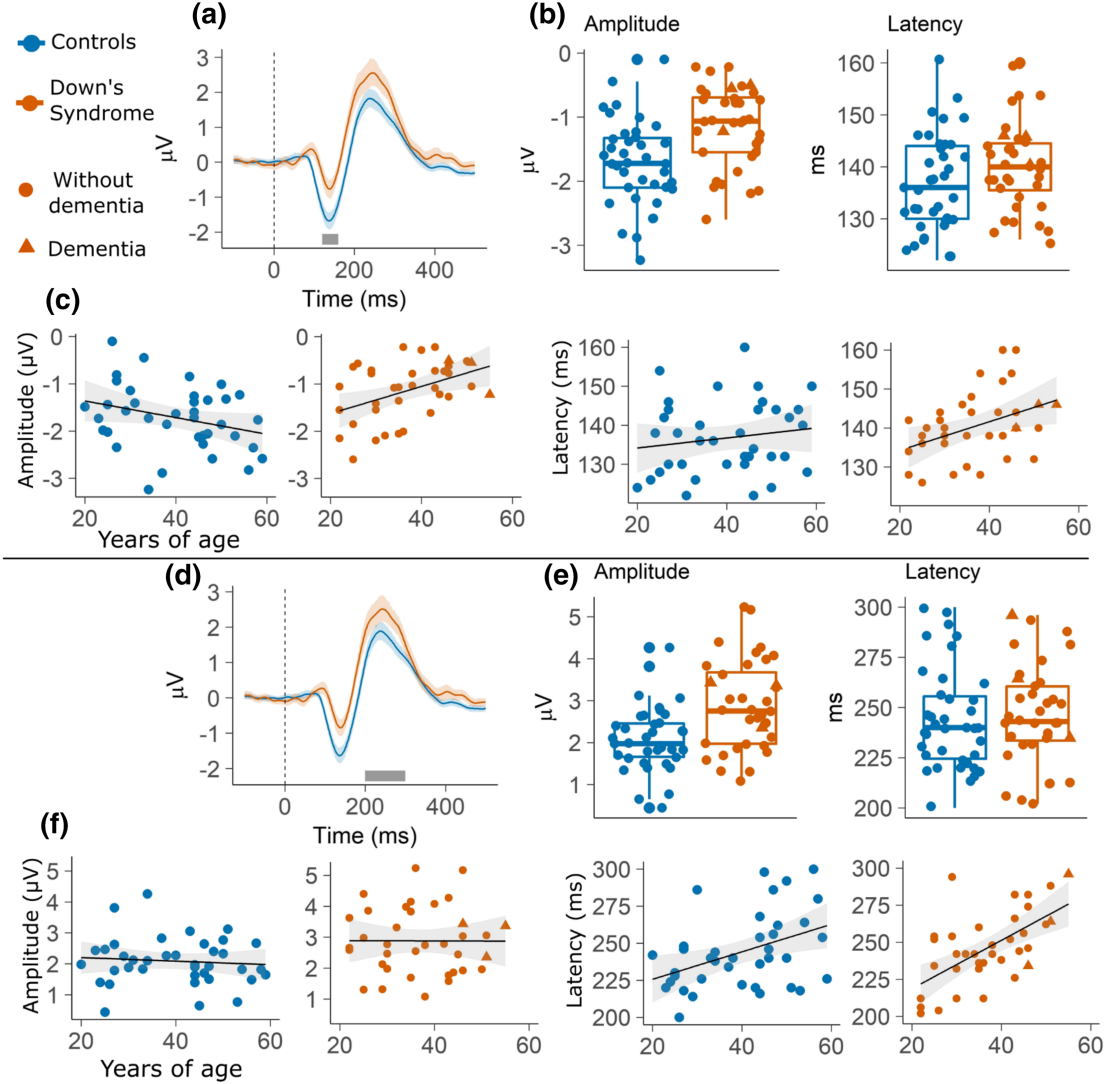
The top panel reports results on the lMMN: (a) Waveforms of controls and participants with DS. The grey segment marks the 120 to 160 ms window in which the peaks have been identified; (b) boxplots contrasting peak amplitude and latency of controls and participants with DS; (c) fitted linear models of peak amplitude and latency as dependent variable and *age* as predictor. The linear models have been plotted separately for the *group* factor so that to illustrate the interaction between *age* and *group* predictors. In all plots, shading represents 95% confidence intervals. The bottom panel reports results on the lP300: (d) Waveforms of controls and participants with DS. The grey segment marks the 200 to 300 ms window in which the peaks have been identified; (e) boxplots contrasting peak amplitude and latency of controls and participants with DS; (f) fitted linear models of peak amplitude and latency as dependent variable and *age* as predictor. The linear models have been plotted separately for the *group* factor so that to illustrate the interaction between *age* and *group* predictors. In all plots shading represents 95% confidence intervals.

The best model was the model with the interaction between *Age* and *Group* (model *d* in Formulae 1). This model was significantly better (*χ* ^2^_1_ = 10.50, *p* = 0.0020, *BF*_db_ = 22.18) than the model with only the main factors (model *c*). Model *d* explained a significant amount of the variance of lMMN amplitude changes with age (*F*_3,70_ = 8.92, *p* = 0.000044, *R* ^2^_Adj_ = 0.25). In the model, *Group* (*B* = −1.19, *SE* = 0.37, *t*_70_ = −2.14, *p* = 0.036), *Age* (*B* = −0.018, *SE* = 0.0088, *t*_70_ = −1.99, *p* = 0.050), and the *Group* and *Age* interaction (*B* = 0.046, SE = 0.01, *t*_70_ = 3.27, *p* = 0.0017) significantly predicted amplitude. The linear model fitted only on TD control data (model *e*, Formulae 1) was not significant and did not differ from the intercept (model *f*, Formulae 1) as confirmed by *BF*_ef_ = 0.96. On the other hand, the model fitted on DS data (model *e*) was significant (*F*_1,34_ = 7.78, *p* = 0.0086, *R* ^2^_Adj_ = 0.16). *Age* significantly predicted amplitude in DS (*B* = 0.029, *SE* = 0.010, *t*_35_ = 2.79, *p* = 0.0086) and the model differed from the intercept (*χ* ^2^_1_ = 7.42, *p* = 0.0065, *BF*_ef_ = 6.81). The log likelihood test on latency showed that model *c* with the main factors was the best model, having a better fit than model *b* (*χ* ^2^_1_ = 4.54, *p* = 0.033, *BF*_cb_ = 1.12). Model *c* explained a significant amount of the variance of lMMN latency (*F*_2,71_ = 4.086, *p* = 0.021, *R* ^2^_Adj_ = 0.078). *Age* (*B* = 0.22, *SE* = 0.10, *t*_71_ = 2.21, *p* = 0.030) and *Group* (*B* = 4.39, *SE* = 2.07, *t*_71_ = 2.12, *p* = 0.038) were significant predictors of latency. Hence, the data showed that latency slowed down as a function of age in both groups. Regressions are plotted in Figure 4 and summarised in Table 2.

**TABLE 2:** lMMN likelihood ratio test results

	Models compared	*χ* ^2^_1_	*p* value	*BF*_10_
	(b) y~Age (a) y~1**	0.23	n.s.	*BF*_ba_ = 0.13
Amplitude	(c) y~Age+Group** (b) y~Age	13.21	≤0.001	*BF*_cb_ = 85.96
	(d) y~Age*Group** (b) y~Age+Group	10.50	≤0.01	*BF*_dc_ = 22.18
	(e) $y_{\mathrm{TD}}\sim {\mathrm{Age}}_{\mathrm{TD}}$ (f) $y_{\mathrm{TD}}\sim 1**$	3.55	n.s.	*BF*_ef_ = 0.96
	(e) $y_{\mathrm{DS}}\sim {\mathrm{Age}}_{\mathrm{DS}}**$ (f) $y_{\mathrm{DS}}\sim 1$	7.42	≤0.01	*BF*_ef_ = 6.81
Latency	(b) y~Age (a) y~1**	3.35	n.s.	*BF*_ba_ = 0.68
	(c) y~Age+Group (b) y~Age**	4.53	n.s.	*BF*_cb_ = 1.12
	(d) y~Age*Group (b) y~Age+Group**	1.43	n.s.	*BF*_dc_ = 0.24

*Note*: The models compared are noted with the letter as in Formulae 1. *χ* ^2^, *p* values, and Bayes factors (*BF*) with the simpler model as denominator are reported. Models were considered statistically significant at the corrected *α* = 0.025. The winning model is marked with a double asterisk.

#### 3.3.2 lP300

Comparing the models that predicted variations in amplitude, the model with *Group* and *Age* (model *c* in Formulae 1) was the best model, providing a significant improvement to the model (model *b* in Formulae 1) with *Age* as the only predictor (*χ* ^2^_1_ = 12.16, *p* = 0.00049, *BF*_cb_ = 50.75). Model *c* explained a significant amount of the variance of lP300 amplitude changes (*F*_2,71_ = 6.74, *p* = 0.0021, *R* ^2^_Adj_ = 0.14). Only the factor *Group* significantly predicted amplitude (*B* = 0.78, *SE* = 0.22, *t*_71_ = 3.56, *p* = 0.000067).

Regarding latencies, the best model was model *b* with only *Age* as predictor, being the winning model over the intercept (model *a*; *χ* ^2^_1_ = 19.89, *p* = 0.000082, *BF*_ba_ = 2420). Model *b* explained a significant amount of the variance of lP300 latency (*F*_1,72_ = 21.97, *p* = 0.00012, *R* ^2^_Adj_ = 0.23) in which *Age* was a significant predictor of latency (*B* = 1.16, *SE* = 0.25, *t*_72_ = 4.71, *p* = 0.00012). Regressions are plotted in Figure 4 and summarised in Table 3.

**TABLE 3:** lP300 likelihood ratio test results

	Models compared	*χ* ^2^_1_	*p* value	*BF*_10_
Amplitude	(b) y~Age (a) y~1**	0.70	n.s.	*BF*_ba_ = 0.17
	(c) y~Age+Group** (b) y~Age	12.16	≤0.001	*BF*_cb_ = 50.75
	(d) y~Age*Group (b) y~Age+Group**	0.07	n.s.	*BF*_dc_ = 0.12
Latency	(b) y~Age** (a) y~1	19.89	≤0.0001	*BF*_ba_ = 2420
	(c) y~Age+Group (b) y~Age**	1.24	n.s.	*BF*_cb_ = 0.22
	(d) y~Age*Group (b) y~Age+Group**	2.02	n.s.	*BF*_dc_ = 0.32

*Note*: The models compared are noted with the letter as in Formulae 1. *χ* ^2^, *p* values, and Bayes factors (*BF*) with the simpler model as denominator are reported. Models were considered statistically significant at the corrected *α* = 0.025. The winning model is marked with a double asterisk.

#### 3.3.3 gMMN

**FIGURE 5:**
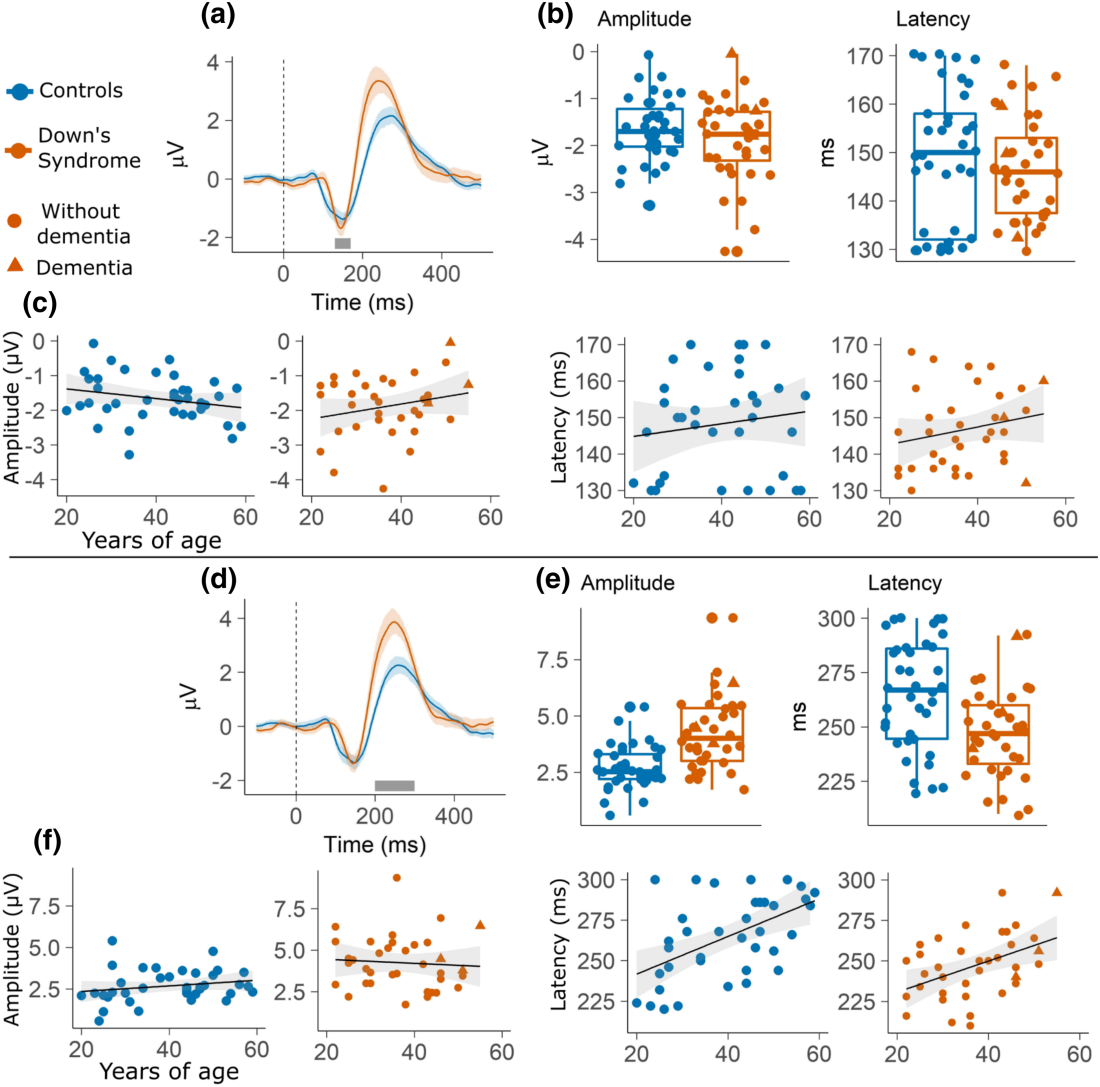
The top panel reports results on the gMMN: (a) waveforms of controls and participants with DS. The grey segment marks the 130 to 170 ms window in which the peaks have been identified; (b) boxplots contrasting peak amplitude and latency of controls and participants with DS; (c) fitted linear models of peak amplitude and latency as dependent variable and *age* as predictor. The linear models have been plotted separately for the *group* factor so that to illustrate the interaction between *age* and *group* predictors. In all plots, shading represents 95% confidence intervals. The bottom panel reports results on the gP300: (d) waveforms of controls and participants with DS. The grey segment marks the 200 to 300 ms window in which the peaks have been identified; (e) boxplots contrasting peak amplitude and latency of controls and participants with DS; (f) fitted linear models of peak amplitude and latency as dependent variable and *age* as predictor. The linear models have been plotted separately for the *group* factor so that to illustrate the interaction between *age* and *group* predictors. In all plots, shading represents 95% confidence intervals.

Comparing the models that predicted variations in amplitudes based on *Age* and *Group*, the model with the interaction between *Age* and *Group* as a predictor (model *d*) was the best model. It provided a significant improvement to the model with only the main factors (model *c* in Formulae 1; *χ* ^2^_1_ = 4.08, *p* = 0.043). However, the *BF* calculated was *BF*_dc_ = 0.89 and the statistics suggest that there is no relationship between the predictors and amplitude (*p* = 0.14). Regarding latencies, no model fitted the data better than the intercept (model *a*). Regressions are plotted in Figure 5 and summarised in Table 4.

**TABLE 4:** gMMN likelihood ratio test results

	Models compared	*χ* ^2^_1_	*p* value	*BF*_10_
Amplitude	(b) y~Age (a) y~1**	0.02	n.s.	*BF*_ba_ = 0.12
	(c) y~Age+Group (b) y~Age**	1.57	n.s.	*BF*_cb_ = 0.25
	(d) y~Age*Group** (b) y~Age+Group	4.08	≤ 0.05	*BF*_dc_ = 0.80
Latency	(b) y~Age (a) y~1**	2.15	n.s.	*BF*_ba_ = 0.34
	(c) y~Age+Group (b) y~Age**	0.11	n.s.	*BF*_cb_ = 0.12
	(d) y~Age*Group (b) y~Age+Group**	0.05	n.s.	*BF*_dc_ = 0.12

*Note*: The models compared are noted with the letter as in Formulae 1. *χ* ^2^, *p* values, and Bayes factors (*BF*) with the simpler model as denominator are reported. Models were considered statistically significant at the corrected *α* = 0.025. The winning model is marked with a double asterisk.

#### 3.3.4 gP300

Comparing the models that predicted variations in amplitudes, the model with *Age* and *Group* as main factors (model *c*) was the best model. It fitted the data better than the previous model (model *b*; *χ* ^2^_1_ = 22.98, *p* = 0.0000016, *BF*_cb_ = 1140). The model was a good fit of the data (*F*_2,71_ = 13.00, *p* = 0.000015, *R* ^2^_Adj_ = 0.25). Only the factor *Group* was a significant predictor (*B* = 1.58, *SE* = 0.31, *t*_71_ = 5.09, *p* = 0.0000029). Regarding latencies, model *c* with both *Age* and *Group* as main factors was the best model. It fitted the data better than the model with only *Age* as predictor (model *b*; *χ* ^2^_1_ = 9.45, *p* = 0.0021, *BF*_cb_ = 13.12). The model explained a significant amount of the variance of gP300 latency (*F*_2,71_ = 18.94, *p* = 0.00000026, *R* ^2^_Adj_ = 0.33) in which *Age* was a significant predictor (*B* = 1.08, *SE* = 0.22, *t*_71_ = 4.79, *p* = 0.0000089), as well as *Group* (*B* = −14.61, *SE* = 4.70, *t*_71_ = −3.11, *p* = 0.0027). Regressions are plotted in Figure 5 and summarised in Table 5.

**TABLE 5:** gP300 likelihood ratio test results

	Models compared	*χ* ^2^_1_	*p* value	*BF*_10_
Amplitude	(b) y~Age (a) y~1**	0.11	n.s.	*BF*_ba_ = 0.12
	(c) y~Age+Group** (b) y~Age	22.98	≤0.0001	*BF*_cb_ = 1140
	(d) y~Age*Group (b) y~Age+Group**	0.94	n.s.	*BF*_dc_ = 0.19
Latency	(b) y~Age** (a) y~1	22.18	≤0.0001	*BF*_ba_ = 762
	(c) y~Age+Group** (b) y~Age	9.45	≤0.01	*BF*_cb_ = 13.12
	(d) y~Age*Group (b) y~Age+Group**	0.20	n.s.	*BF*_dc_ = 0.13

*Note*: The models compared are noted with the letter as in Formulae 1. *χ* ^2^, *p* values, and Bayes factors (*BF*) with the simpler model as denominator are reported. Models were considered statistically significant at the corrected *α* = 0.025. The winning model is marked with a double asterisk.

### 3.4 Demographics at T2

One year after the initial assessment, no participant transitioned to an AD diagnosis at the CAMDEX‐DS. Table 6 provides more demographic and cognitive detail for the cognitive follow‐up of participants.

**TABLE 6:** Demographics and CAMCOG scores of the participants in the cognitive follow‐up phase

	Min	Max	Mean	*SD*
T1 age (years)	22	55	37.0	9.3
T1 total CAMCOG score	58	105	83.4	14.0
T2 total CAMCOG score	48	104	81.4	15.1
T2‐T1 total CAMCOG score	‐17	5	−2.0	4.7

*Note*: T1 represents time 1 (initial assessment), T2 represents time 2 (follow‐up assessment). *SD* is standard deviation from the mean.

### 3.5 Cognitive decline in DS

**FIGURE 6:**
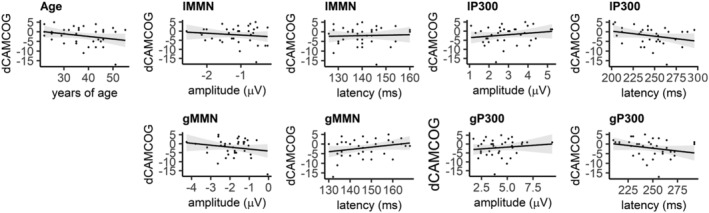
Correlation between total dCAMCOG score and age (first plot). Linear models with amplitude and latency of each ERP components as predictors and total dCAMCOG score as dependent variable. Shading represents 95% confidence intervals.

Scores to the CAMCOG at T2 were significantly lower than scores at T1 (*t*_34_ = 2.51, *p* = 0.017, Cohen's *d* = 0.42). The Spearman rank‐order correlation between dCAMCOG and age was not significant (*ρ*_33_ = −0.16, *p* = 0.36, *BF*_10_ = 1.07). Therefore, age was considered no further in the following analyses. The 34 participants' total CAMCOG difference scores ranged from −17 to 5 points with a mean change of −2.06 points (*SD* = 4.77 points). Results are plotted in Figure 6.

#### 3.5.1 lMMN

Sequentially comparing the models reported in Formulae 2, no model was a significant improvement over the preceding model in models with amplitude as predictor (*BF*_ba_ = 0.23, *BF*_cb_ = 0.17, *BF*_dc_ = 0.17, *BF*_de_ = 0.19) nor in models with latency as predictor (*BF*_ba_ = 0.17, *BF*_cb_ = 0.17, *BF*_dc_ = 0.18, *BF*_de_ = 0.17). This means that no association was found between the latency of the lMMN and cognitive decline and also no relationship was found between the amplitude of the lMMN and cognitive decline.

#### 3.5.2 lP300

No model was a significant improvement over the preceding model in models with amplitude as predictor (*BF*_ba_ = 0.31, *BF*_cb_ = 0.17, *BF*_dc_ = 0.23, *BF*_de_ = 0.17) nor using models with latency as predictor (*BF*_ba_ = 0.59, *BF*_cb_ = 0.17, *BF*_dc_ = 0.58, *BF*_de_ = 0.17). This means that no association was found between the latency and amplitude of the lP300 and cognitive decline.

#### 3.5.3 gMMN

No model was a significant improvement over the preceding model neither when amplitude was the predictor (*BF*_ba_ = 0.35, *BF*_cb_ = 0.17, *BF*_dc_ = 0.39, *BF*_de_ = 0.17) nor when latency was the predictor (*BF*_ba_ = 0.63, *BF*_cb_ = 0.18, *BF*_dc_ = 0.20, *BF*_de_ = 0.17). Therefore, no association was found between the latency and amplitude of the gMMN and cognitive decline.

#### 3.5.4 gP300

No model was a significant improvement over the preceding model when amplitude was the predictor (*BF*_ba_ = 0.24, *BF*_cb_ = 0.17, *BF*_dc_ = 0.23, *BF*_de_ = 0.17). When latency was the predictor no model was a significant improvement over the preceding one (*BF*_ba_ = 0.52, *BF*_cb_ = 0.17, *BF*_dc_ = 0.89, *BF*_de_ = 5.37). Therefore, also for gP300 no association was found between latency or amplitude and cognitive decline.

## 4 DISCUSSION

We aimed at characterising ERPs of auditory predictive processes in the DS population and assessing their potential as electrophysiological markers of pathological ageing and cognitive decline in this population. In a case–control study, participants with DS and TD age‐matched controls were presented sequences of five tones that could either consist of five identical tones (*local standard* sequences) or four identical tones and a fifth different tone (*local deviant* sequences). Furthermore, within each experimental block some sequences were frequent (*global standard* sequences) and others were rare (*global deviant* sequences). Contrasting standard and deviant sequences within the two levels of violation hierarchy generated ERP components that reflected respectively local and global effects of auditory expectation violation. To evaluate whether such components were a predictor of cognitive decline in DS, the CAMCOG‐DS was administered to DS participants at the same time as the EEG recording (T1) and then scores were gathered again 10 to 14 months later (T2). The predictive power of the ERPs over cognitive decline was quantified by the relationship between the ERPs at T1 and the CAMCOG difference score between the two time points (T2 minus T1). Surprisingly little research has been conducted to characterise ERPs as a means for detecting auditory regularity violation in the DS population. Therefore, we implemented an explorative method to characterise the timing and spatial distribution of ERPs generated by contrasting standard to deviant stimuli in DS.

Detection of local deviancy appeared to be preserved in individuals with DS. The effect emerged as the classical lMMN/lP300/RON complex that represents automatic change detection and reorienting of attention (Horváth et al., 2008; Justo‐Guillén et al., 2019; Schröger et al., 2000). Topography and timing of these components were similar in both groups with the difference that participants with DS had a reduced lMMN and enhanced lP300 response. Furthermore, a frontocentral negative deflection appeared in both groups after 400 ms. Such deflection could be identified as the RON that marks the cognitive recovery from distraction and that often follows the lMMN‐lP300 complex (Berti, 2008; Berti & Schröger, 2003; Justo‐Guillén et al., 2019). The late response to local deviancy was very similar between groups with a slight reduced positivity in DS (Figure 2).

In a predictive coding framework, the reduced lMMN response suggests a preserved yet attenuated signalling of sensory prediction error in DS. Contrary to our hypotheses, the following P300 component was enhanced in this group. This was surprising in light of a recent study whose results suggest a delayed frontal lP300 in the ageing population (Hsu et al., 2021). Little is known on the P300 is DS, let alone in the context of detection of local–global violations. A study assessing the P300 is DS found a frontal shift compared with TD controls (Kakigi et al., 1994) and suggested that it might represent an unusual reaction to unpredictable shifts in pitch. The frontocentral P300 has been linked to the involuntary reorienting of attention caused by distracting stimuli (Kaipio, 2016; Masson & Bidet‐Caulet, 2019; Polich & Criado, 2006; Wetzel & Schröger, 2007), and in some cases, it has been associated with poorer behavioral performance (Berti & Schröger, 2003; Wetzel et al., 2006). It has also been suggested to represent the physical and emotional arousal state induced by unpredictability (Delplanque et al., 2005; Masson & Bidet‐Caulet, 2019). The reduced efficiency in signalling prediction error in DS may increase the unpredictability valence of deviant stimuli, triggering a stronger involuntary reorienting of attention.

Detection of global deviancy was also preserved in DS. The violation of global rules elicited a gMMN/gP300/RON complex that was similar to that elicited in TD controls. Again contrary to our hypotheses, the gP300 was more positive in DS and had shorter latencies than in TD. Interestingly, the gP300 faded quicker in DS, disappearing around 300‐350 ms while in TD controls it continued up to 400 ms (Figure 3). The RON appears to be delayed in DS with maximum difference from TD controls at 500–550 ms (Figure 3), possibly reflecting reduced efficiency in recovering from distraction. The results show that the understanding and processing of global rules and statistical regularities is present in DS but attention‐dependent components appear to be altered compared with TD controls. Earlier latency and shorter gP300 time course may again be interpreted as a more prevalent automatic redirection of attention compared with TD controls, even when the paradigm requires to understand global rules. The interpretation is for now a suggested hypothesis as the data reported slightly deviate from the traditional global effect (Chennu et al., 2013, 2016; Chennu & Bekinschtein, 2012; Hsu et al., 2021). To the expert eye it surely has stood out the lack of parietal P300 response to global deviancy, even in TD controls. We can confidently explain this difference as the result of the instructions given to participants. The parietal P300 is elicited by target stimuli, while the frontal P300 is elicited also by nontarget stimuli (Polich, 2007; Polich & Criado, 2006). In our study participants were asked to simply pay attention to stimuli by reporting which sequences were common and which were uncommon. On the other hand, the traditional global–local paradigm requires the participant to count uncommon sequences. Hence, in the latter scenario uncommon sequences are targets, while in the case of our study they were not. This explains the lack of parietal P300 in both groups. The decision of giving simpler instructions came from the practical experience of our lab in working with participants with DS, who can get easily overwhelmed in experimental settings and that may struggle in understanding verbal instructions. This may be a limitation of the study due to the characteristic of the population of interest. Nevertheless, our study shows that the frontal P300 can be a marker of higher level prediction error, and that the detection of long‐term stimulus deviance is preserved in DS.

The second aim of our study was to assess whether electrophysiological markers of auditory violation may be a marker of ageing in DS. Individuals with DS experience premature ageing and AD pathology affects nearly the entirety of the DS population by the time they reach their 60s (Grothe et al., 2017; Sperling et al., 2013; Teipel et al., 2020). Individuals with DS have underdeveloped frontal lobe (Holland et al., 1998; Lautarescu et al., 2017) and early frontal neurodegeneration (Annus et al., 2016). The latter may also explain early onset of executive dysfunction in DS with AD. Therefore, frontal components are of particular interest for tracking ageing and cognitive decline in DS. The models predicting lMMN amplitude as a function of age and group showed that amplitude decreases with age in DS but not in TD controls. The results may reflect impaired pre‐perceptual change detection (Cheng et al., 2013; Näätänen et al., 2007, 2014). Alternatively in the context of a predictive coding framework, they may indicate that the ability to make accurate statistical predictions about the incoming stimuli decreases with age in people with DS but not in age‐matched controls (Friston, 2003, 2005; Garrido, Kilner, Kiebel, & Friston, 2009). According to models of the generation of the lMMN (Friston, 2003, 2005; Garrido et al., 2008; Garrido, Kilner, Kiebel, & Friston, 2009; Garrido, Kilner, Stephan, & Friston, 2009), the prediction error represented by the lMMN is dependent on both backward (top‐down) and forward (bottom‐up) connections between the levels of the network hierarchy. If the decrease of the brain's predictive power is what best explains lMMN reductions in people with DS, further investigation of how age‐related decay of the lMMN response in people with DS affects these two directions of connectivity is indicated. Future studies could aim at directly testing the two alternative frameworks of the lMMN within the context of DS and ageing. Regarding the P300, it has been found to decrease in amplitude and increase in latency with normal ageing, and to be a marker of AD (Cecchi et al., 2015; Polich et al., 1990; Polich & Corey‐Bloom, 2005). Latency of both the lP300 and gP300 were predicted by age. While group differences in gP300 latency were apparent already in young participants, the relationship between lP300/gP300 and age was similar in both groups.

While the amplitude of the lMMN was predicted by age in DS, the same was not the case for TD controls. Notably, while the main factor *Age* was significant when it was the only predictor of the lMMN amplitude, the *BF* of 0.68 showed that the model cannot be taken as conclusive evidence of age‐related lMMN modulation. Some research showed that the MMN response decreases with age, suggesting a decline in sensory memory, perceptual accuracy and the brains' predictive power (Cheng et al., 2013; Horváth et al., 2007; Kiang et al., 2009; Näätänen et al., 2011; Schiff et al., 2008). Rather, our results are in agreement with a previous study which found intact lMMN in seniors (Hsu et al., 2021). Discrepancies in findings between studies assessing the MMN in the ageing population may also be due to the demographics of the cohorts. In our study, people with DS and controls had an average age of 36.8 and 39.8 years, respectively, while studies assessing the lMMN in elderly controls typically have older participants. It may be that age‐related decay in our control cohort was not sufficient to be reflected in changes in the MMN. This is consistent with the hypothesis that at 40 years of age, DS begin to show abnormal Aβ binding in the brain compared with controls (Annus et al., 2016). A second interpretation is that lMMN decay with age reflects characteristic intrinsic to DS. To further address this issue, we compared the two groups only including those under 40s. We did not find any difference in lMMN amplitude in those under 40s, which might suggest that the effect of ageing is in this study specific to DS. However, Bayesian comparisons provided only anecdotal evidence for the null hypothesis. Overall, we provide evidence that in the case of DS the amplitude of the lMMN decreases with age. Whether it represents accelerated ageing or pathological ageing mixed with intrinsic properties of DS pathology remains to be further investigated.

It is important to note that the cohort of DS participants included three individuals with AD. The decision to keep those participants in our sample was driven by the strong evidence that people with DS will invariantly develop AD during their lifespan (Holland et al., 1998; Lautarescu et al., 2017). The presence of AD is typically considered a dichotomous variable used as inclusion or exclusion criteria. Instead, we made the theoretical decision of treating it as a continuous variable that simply expresses a degree of progression of inevitable pathological ageing in individuals with DS. As apparent by the visual representation of the data (Figures 4 and 5), the AD participants fell within the normal range of the rest of the DS group.

Our data showed that in DS, none of the ERPs measures could predict cognitive decline 1 year later. Results from the case–control study suggested that the lMMN is a measure sensitive to ageing associated with DS; however, the relationship with cognitive decline as measured by cognitive tests is less clear. First, there was no correlation between dCAMCOG and age. Second, there was no evidence for any relationship between lMMN measures and cognitive decline as measured through the CAMCOG‐DS, with *BF* ranging from 0.17 and 0.23 pointing towards substantial evidence for the null hypothesis. The contrast between scores at T1 and T2 showed that the CAMCOG was able to detect a significant cognitive decline after 12 months, although with a small effect size. Hence, one explanation of a lack of relationship between lMMN and dCAMCOG may be that the extent of cognitive decline was not big enough to be reflected in lMMN variations. One reason for this may be that the time gap between T1 and T2 might not have been long enough to see a statistically significant effect. Alternatively, the study might be underpowered to detect potentially small effects. It is also important to keep in mind that the CAMCOG‐DS assesses a variety of complex cognitive functions beyond perceptual and predictive processes (Fonseca et al., 2018; Huppert et al., 1996; Ter Horst et al., 1993). Hence, the complexity of the CAMCOG‐DS scores may overshadow any decline in the processes reflected by the lMMN.

An important aspect of this research is that it brings a first evidence that a relatively affordable, flexible and non‐invasive technique such as EEG may be used in conjunction to change detection paradigms in populations with intellectual disabilities. These populations may find experimental settings stressful and may find it challenging to comply with requests such as maintaining sustained attention or inhibit movements. Being able to detect pathology by analysing attention‐independent components and the statistical tools that have been developed to correct for muscle activity can be very valuable for research in the field of intellectual and developmental disabilities.

There are some limitations to this study. First, the instructions given to the participants deviate from the standard instructions usually given during the global–local paradigm. As we already discussed, the decision had been made on the basis of the experience our laboratory had with testing participants with DS in experimental settings. However, this made it difficult to compare out results of the global effect with previous literature. A second limitation is that there was no EEG measurement at T2. Future research should aim at filling this gap. The degree of lMMN variation over time in DS within individual participants should be assessed. If the lMMN is a potential tool to measure ageing in the DS population, it is clinically important to establish its temporal sensitivity. A third limitation is that 1 year gap between T1 and T2 might have been insufficient. The lMMN at T1 may not be predictive of cognitive decline after 12 months but a change in lMMN amplitude over time may be predictive of future cognitive decline on a larger timescale. Finally, TD controls were not retested at T2. DS falls under the umbrella of intellectual disabilities and therefore the decision to use the CAMCOG‐DS was made as it is a specific measure of cognitive decline in DS and it has been validated specifically in DS. To this date, there is no data available showing that the CAMCOG and the CAMCOG‐DS are comparable. Furthermore, the CAMCOG has been validated for individuals over 65 years of age (Roth et al., 1986), which is above the age range of the age‐matched TD control group in the present study. Therefore, the comparisons between groups in terms of relationship between ERPs and cognitive decline would have had to be made on the basis of different measures.

## 5 CONCLUSIONS

The present study investigated the detection of local and global prediction of auditory violation in DS and investigated how ERPs could be potentially means to study pathological ageing process in DS. We showed that, detection of auditory irregularities is preserved in DS both at the local and global level. On the other hand, the time course and intensity of these effect is affected by DS pathology.

We also showed that age predicted the amplitude of the lMMN in DS. The amplitude decreased with increasing age. On the other hand, in the TD group age did not predict lMMN amplitude. This suggests that in people with DS the lMMN could be a marker to assess the impairment of processes and structures associated with the generation of lMMN responses.

The study also investigated the ERPs of auditory change detection as predictors of cognitive decline during 12 months' time. Overall the data did not provide evidence in support of a significant relationship between ERPs and cognitive decline in DS as measured by the CAMCOG‐DS.

**Abbreviations**
ACE‐R: Addenbrookes Cognitive Examination‐Revised
AD: Alzheimer's disease
Aβ: beta‐amyloid
*BF*_ij_
: Bayes factor where *i* is the null and *j* the alternative model
CAMCOG‐DS: The Cambridge Cognitive Examination for Older Adults with Down's Syndrome
CAMDEX‐DS: The Cambridge Examination for Mental Disorders of Older People with Down's Syndrome and Others with Intellectual Disabilities
dCAMCOG: CAMCOG difference score
DS: Down's syndrome
EEG: Electroencephalography
ERP: event‐related potential
gMMN: global mismatch negativity
gP300: global P300
ICA: independent component analysis
JDR: Join Dementia Research
KBIT‐2: Kaufman Brief Intelligence Test, Second Ed.
lMMN: local mismatch negativity
lP300: local P300
M: mean
PIB: Pittsburgh Compound B
SD: standard deviation
T1: time 1
T2: time 2
TD: typically developing

## CONFLICT OF INTEREST

The authors have no conflicts of interest to declare.

## AUTHOR CONTRIBUTION

**Conceptualization and design**: SRJ, TAB, VN, SC, AJH, SHZ, HR. **Data acquisition:** SRJ, MJW. **Data analysis:** CA, SRJ. **Drafting:** CA, SRJ. **Figures:** CA. **Supervision:** SC, VN, TAB, HR. **Review:** CA, AL, TAB, VN, AJH, ICHC. **Funding:** AJH, SHZ, HR, SRJ. CA and SRJ contributed equally to the paper.

### PEER REVIEW

The peer review history for this article is available at https://publons.com/publon/10.1111/ejn.15762.

## Data Availability

The data that support the findings of this study are available from the corresponding author upon reasonable request.
